# The XRE Family Transcriptional Regulator SrtR in *Streptococcus suis* Is Involved in Oxidant Tolerance and Virulence

**DOI:** 10.3389/fcimb.2018.00452

**Published:** 2019-01-10

**Authors:** Yuli Hu, Qian Hu, Rong Wei, Runcheng Li, Dun Zhao, Meng Ge, Qing Yao, Xinglong Yu

**Affiliations:** College of Veterinary Medicine, Hunan Agricultural University, Changsha, China

**Keywords:** comparative genomics, adaptation, stress response, regulation, reverse mutation

## Abstract

*Streptococcus suis* is a zoonotic pathogen that harbors anti-oxidative stress genes, which have been reported to be associated with virulence. Serial passage has been widely used to obtain phenotypic variant strains to investigate the functions of important genes. In the present study, *S. suis* serotype 9 strain DN13 was serially passaged in mice 30 times. The virulence of a single colony from passage 10 (SS9-P10) was found to increase by at least 140-fold as indicated by LD_50_ values, and the increased virulence was stable for single colonies from passage 20 (SS0-P20) and 30 (SS0-P30). Compared to the parental strain, the mouse-adapted strains were more tolerant to oxidative and high temperature stress. Genome-wide analysis of nucleotide variations found that reverse mutations occurred in seven genes, as indicated by BLAST analysis. Three of the reverse mutation genes or their homologs in other bacteria were reported to be virulence-associated, including *ide*_*Ssuis*_ in *S. suis*, a homolog of *malR* of *Streptococcus pneumoniae*, and a homolog of the prepilin peptidase-encoding gene in *Legionella pneumophila*. However, these genes were not involved in the stress response. Another gene, *srtR* (stress response transcriptional regulator), encoding an XRE family transcriptional regulator, which had an internal stop in the parental strain, was functionally restored in the adapted strains. Further analysis of DN13 and SS9-P10-background *srtR*-knock-out and complementing strains supported the contribution of this gene to stress tolerance *in vitro* and virulence in mice. *srtR* and its homologs are widely distributed in Gram-positive bacteria including several important human pathogens such as *Enterococcus faecium* and *Clostridioides difficile*, indicating similar functions in these bacteria. Taken together, our study identified the first member of the XRE family of transcriptional regulators that is involved in stress tolerance and virulence. It also provides insight into the mechanism of enhanced virulence after serial passage in experimental animals.

## Introduction

*Streptococcus suis* is an economically important opportunistic pathogen that can cause septicemia, meningitis, and arthritis, among other diseases in swine. *S. suis* serotypes 2 and 9 are two of the most predominant serotypes in pig farms worldwide. In some European countries such as Spain and the Netherlands, serotype 9 is the most prevalent (Goyette-Desjardins et al., 2014). This serotype is also frequently isolated from diseased pigs in China (Wu et al., 2008; Dong et al., 2017). More importantly, humans can also be infected by *S. suis* serotype 9 after the ingestion of contaminated pork-derived food (Kerdsin et al., 2015), indicating that it is a zoonotic pathogen.

Reactive oxygen species such as hydroxyl radical (^.^OH), hydrogen peroxide (H_2_O_2_), and superoxide (^.^O_2_) are important components of the host defense mechanism to clear microorganisms (Rada and Leto, 2008; Lam et al., 2010). Bacterial pathogens have developed complex oxidative stress resistance mechanisms including multiple enzymes to clear reactive oxygen species and protein-binding to free iron to block the transformation of H_2_O_2_ to highly toxic ^.^OH and OH^−^ species via the Fenton reaction (Imlay, 2008). These anti-oxidant systems help to protect bacterial DNA, proteins, and lipids from damage and to repair biological molecules (Tsou et al., 2008; Ezraty et al., 2017), facilitating survival in the host (Tang et al., 2012). Furthermore, co-ordinate regulation of the oxidative stress response and virulence factors or virulence-associated genes by transcriptional regulators has been reported in a variety of bacteria (Verneuil et al., 2005; Chen et al., 2009; Lebreton et al., 2012; Reen et al., 2013; Zheng et al., 2014). For example, upon exposure to oxidative stress, the regulator AsrR in *Enterococcus faecium* is inactivated by changes in conformation caused by cysteines oxidation, and released from the promoters of some adhesin-encoding genes, leading to up-regulation of these genes and the promotion of adhesion to epithelial cells, thereby facilitating the establishment of infection (Lebreton et al., 2012).

Fever caused in response to infection is thought to be a defense mechanism of the host that functions by enhancing the response of immune cells (Evans et al., 2015). On the other hand, the growth of some bacterial species including *S. suis* can be impaired at relatively high temperature (Zhu et al., 2014). Therefore, temperature shifts during infection seem to have a deleterious effect on the survival of bacteria. Thus, thermal tolerance might facilitate survival of pathogens in the host, even though direct evidence of tolerance to high temperature contributing to virulence is lacking.

Microorganisms have also developed physical barriers, such as biofilm to counter the immune system during the infection. Biofilms are aggregates produced by microbes that are usually located at a solid–liquid interface and are encased in a protective extracellular polymeric matrix. Biofilms formed by bacteria can help them to colonize the tissue surface and confer protection against the host defense system or antimicrobial agents (Flemming and Wingender, 2010). This might be one of the reasons that biofilm formation is positively correlated with virulence for some pathogens.

Virulence of some bacterial species in a new host might increase after serial passage in the animal owing to adaptation and evolution (Ebert, 1998). In this process, phenotypic changes can be observed. Combined with omics methods, the relationship between phenotypes and genes in the microbe can be established, shedding light on microbe–host interactions (Hu et al., 2014).

In the present study, strain DN13 of *S. suis* serotype 9 (SS9) was selected as a model for serial passage in mice to obtain highly virulent strains. After 10 serial passages, the virulence of a single isolate from passage 10 (SS9-P10) was found to be increased by at least 140-fold as indicated by LD_50_ values, and this was similar to that of a single isolate from passages 20 (SS9-P20) and 30 (SS9-P30). Oxidative and high temperature stress tolerance assay, as well as biofilm formation assay showed that the mouse-adapted strains (SS9-P10, SS9-P20, and SS9-P30) were more tolerant to oxidative and high temperature stress, and the ability of biofilm formation was increased. Whole genome sequencing revealed that *srtR* (stress response transcriptional regulator), encoding an XRE family transcriptional regulator, which contains a premature stop between the DNA-binding domain and the uncharacterized DUF3955 domain in the parental strain, harbored a reverse mutation after mouse passage. Further analysis of DN13 and SS9-P10-background *srtR* deletion mutant and complementing strains confirmed that this gene is involved in oxidative and high temperature stress tolerance and virulence in mice, but not biofilm formation.

## Materials and Methods

### Ethics Statement

The animal experiment in this study was carried out in accordance with the principles of the Basel Declaration and recommendations of the ARRIVE guidelines of the National Institutes of Health (NIH Publications No. 8023, revised 1978). The protocol was approved by the local Ethics Committee (Review Committee for the Use of Animal Subjects of Hunan Agricultural University).

### Bacterial Strains and Culture Conditions

Streptococcal strains and plasmids used in this study are listed in Table 1. The SS9 strain DN13 was isolated from a pig with bacteremia at Chenzhou city, Hunan province in 2013 and was previously identified as serotype 9 by sero-specific PCR (Kerdsin et al., 2014). Bacteria were cultured in brain heart infusion (BHI) at 37°C or were streaked onto BHI agar and incubated at 37°C unless otherwise mentioned. Liquid Luria broth or agar was used for the culture of *Escherichia coli* strains. Antibiotics were added to the media as required at the following concentrations: 50 μg/ml for spectinomycin for both *S. suis* and *E. coli*; 8 and 37 μg/ml of chloramphenicol for *S. suis* and *E. coli*, respectively.

**Table 1:** Bacterial strains and plasmids used in this study.

**Bacterial strains**	**Description**	**Source or reference**
DN13	Isolated from diseased pigs, *S. suis* serotype 9, mrp^−^ef^−^sly^−^, ST243	Our laboratory
SS9-P10	DN13 serially passaged 10 times in mouse	This study
SS9-P20	DN13 serially passaged 20 times in mouse	This study
SS9-P30	DN13 serially passaged 30 times in mouse	This study
SS9-P10:ΔsrtR	SS9-P10-background *srtR* deletion mutant, Cm^R^	This study
SS9-P10:CΔ srtR	SS9-P10:ΔsrtR complemented with *srtR*; Cm^R^, Spc^R^	This study
DN13-srtR	DN13 complemented with *srtR*; Spc^R^	This study
DN13:ΔsrtR	DN13-background *srtR* deletion mutant, Cm^R^	This study
DN13:CΔsrtR	DN13:ΔsrtR complemented with *srtR*; Cm^R^, Spc^R^	This study
DN13-pSET2	DN13 with empty pSET2 plasmid; Spc^R^	This study
DN13-pSET2-PF0	For determining DN13-derived A6M16_09815 promoter activity in *S. suis*; Cm^R^, Spc^R^	This study
DN13-pSET2-	For determining SS9-P10-derived A6M16_09815 promoter activity in *S. suis*;	This study
PF10	Cm^R^, Spc^R^	
*E. coli* DH5α	Host cell for plasmid manipulating	TSINGKE
**Plasmids**		
pSET1	*E. coli*-*S. suis* shuttle cloning vector; Cm^R^	Takamatsu et al., 2001a
pSET2	*E. coli*-*S. suis* shuttle cloning vector; Spc^R^	Takamatsu et al., 2001a
pSET4s	Thermosensitive suicide vector for construction isogenic mutant in *S. suis*; Spc^R^	Takamatsu et al., 2001b
pSET4s-srtR	For construction of *srtR* deletion mutant	This study
pSET2-srtR	For construction of *srtR* complementing strains; Spc^R^	This study
pSET2-PF0	For determining DN13-derived A6M16_09815 promoter activity; Cm^R^, Spc^R^	This study
pSET2-PF10	For determining SS9-P10-derived A6M16_09815 promoter activity; Cm^R^, Spc^R^	This study

*Cm^R^, chloramphenicol resistance*;

*Spc^R^, spectinomycin resistance*.

### Serial Passage of Bacteria in Mice

Before mouse passage experiments, virulence was evaluated by injecting approximately 10^7^ colony forming units (CFUs; estimated by cell counting on agar) of the DN13 strain in 100 μl of sterile PBS (phosphate-buffered saline) into five 3–4-week old ICR (Institute Of Cancer Research) female mice via intraperitoneal injection. Mice subsequently exhibited depression and anorexia but recovered 4 days post-challenge. Therefore, 10^7^ CFU/mouse was chosen as an initial dose for subsequent challenges. For the first passage, three mice were injected with 50 μl of bacterial suspension containing approximately 10^7^ CFUs via the tail vein. Blood was collected 24 h post-challenge and combined. The mixture was then spread onto chocolate agar. After an 18-h incubation, bacterial lawns were scraped into sterile PBS. After centrifugation at 500 × *g* for 3 min to precipitate the agar pellets, 50 μl of the resultant suspension was used to inoculate the other three mice for the next passage. After passage 10, bacteria used for passage were recovered from the mouse blood and the challenge route was changed to intraperitoneal injection. In detail, the blood of three mice was collected 24 h post-challenge and mixed with sterile PBS with a ratio of 2:7. After centrifugation at 500 × *g* for 3 min to precipitate the blood cells, another three mice received 200 μl of the resultant supernatant via intraperitoneal injection. For mouse passage experiments, SS9 was cultured in tryptone soy broth supplemented with 10% fetal serum or chocolate agar.

### Growth Curve

Overnight *S. suis* cultures were diluted with fresh BHI to an OD_600_ of 0.1 and the dilutions were cultured at 37°C. The OD_600_ was monitored at 1-h intervals for 12 h to determine the growth characteristics.

### Determination of the 50% Lethal Dose (LD_50_) of Mouse-Adapted Strains

SS9-P10, SS9-P20, and SS9-P30 bacteria were overnight cultured in fresh BHI and subcultures of these strains were grown for 4 h to mid-logarithmic phase. After centrifuging and washing with sterile PBS twice, suspensions were 10-fold serially diluted to approximately 10^3^–10^8^ CFU/ml. Each dilution of 100-μl SS9-P10, SS9-P20, and SS9-P30 dilutions was intraperitoneally injected into a group of mice (*n* = 5). Survival of these mice was observed and recorded daily for 12 days post-infection. The SPSS probit analysis program (SPSS 17.0, USA) was used to calculate the LD_50_ (Finney, 1971).

### *In vitro* Stress Experiments

#### Oxidative Stress

To compare oxidative stress responses in the parental and adapted strains or their derivative strains, hydrogen peroxide exposure experiments were performed as previously described (Zhu et al., 2014) with minor modifications. Briefly, 1 ml of cells was harvested at late log-phase (OD_600_ = 0.6, approximately 10^9^ CFU/ml), washed twice with sterile PBS and then suspended in 1 ml of 0.1 M H_2_O_2_ or sterile PBS as a control. After letting stand for 20 min with gentle shaking at 5-min intervals, bacteria were serially 10-fold diluted with sterile PBS six times and an aliquot of 100 μl of diluted bacterial suspensions was plated onto BHI agar for cell counting. The ratio of CFUs in the H_2_O_2_-treated group to that in the control group was calculated as the survival percentage.

#### Temperature Stress

The viability of streptococcal strains at 42°C was examined to evaluate high temperature tolerance. Briefly, late log-phase bacteria were diluted with sterile PBS and spread onto agar. After incubation at 37°C (as the control) and 42°C for 24 h, CFUs were counted to calculate the percent survival at 42°C. All stress experiments were conducted in duplicate and repeated three times.

### Biofilm Formation Assay

Biofilm formation of DN13 and its derivative strains was determined as previously described (Meng et al., 2011). Overnight cultures were diluted with fresh BHI to an OD_600_ of 0.1, and 2 ml of the diluted culture was then transferred to 24-well polystyrene plates for incubation at 37°C. An aliquot of 2 ml of fresh BHI was added to the plates as the blank control. After 3 days of incubation, media were discarded, and planktonic bacteria were removed by washing with sterile PBS twice. Bacteria attached to wells were then fixed with 500 μl of methanol for 30 min and air-dried. Biofilms were stained with 500 μl of 0.1% crystal violet for 30 min at room temperature. Subsequently, unbound dye was washed with tap water twice and plates were dried for 2 h at 70°C. Dyes absorbed to the biofilm were released with 500 μl of 33% (vol/vol) glacial acetic acid. After shaking for 30 min, 200 μl of this sample was transferred to a 96-well microplate and the OD was read at 595 nm.

### Survival in Mouse Whole Blood

Mouse blood was collected from specific pathogen-free ICR mice and mixed with 0.4% sodium citrate for *S. suis* killing *in vitro*, in accordance with a previously described method (de Buhr et al., 2014). Briefly, late log-phase bacteria were diluted with fresh BHI to an OD_600_ of 0.1 and 100 μl of this dilution was added to 900 μl of mouse whole blood containing 0.4% sodium citrate. The viable cells were calculated by cell counting after 0 and 120 min of incubation at 37°C. The ratio of CFUs in the 120-min incubation group to that in the 0-min incubation group was calculated as the survival percentage.

### Antimicrobial Susceptibility Testing

The Kirby–Bauer test was used to compare the drug sensitivity of DN13 and its passages to a series of clinical drugs used in the swine industry as previously described (Dee et al., 1993). Briefly, late log-phase cells were diluted with fresh BHI to 1 × 10^8^ CFU/ml and each 100-μl bacterial dilution was spread onto BHI agar. Subsequently, commercially available drug disks (Hangzhou microbial reagent, China) were placed on the agar. After incubation for 8 h, zone diameters of inhibition were measured.

### Whole Genome Sequencing and Resequencing

Genomic DNA for whole genome sequencing or resequencing was extracted with the Bacterial Genomic DNA Miniprep Kit (AxyPrep™, USA) according to the manufacturer's instructions. The genome of the DN13 parental strain was sequenced at Shanghai Personal Biotechnology Co., Ltd (China). Two libraries, inserting 450 bp and 10 kb, respectively, were constructed. The former was sequenced using the Illumina Miseq platform (Illumina, USA) in Paired-end, 2 × 251 bp mode and the latter was sequenced using the Pacbio RS II platform (Pacific Biosciences, USA) in standard mode. After quality control based on filtering with FastQC^1^, reads obtained by next generation sequencing were assembled using Newbler software (v2.3) after Kmer adjustment resulting in contigs and scaffolds. Pacbio RS II-produced reads were assembled by Celera Assembler (Berlin et al., 2015) to output scaffolds, which were further assembled with PBjelly software (English et al., 2012). Co-linearity analysis was performed using contigs obtained from both libraries to confirm the assembled sequences and location of the contigs.

Whole genome resequencing of SS9-P10, SS9-P20, and SS9-P30 was performed at Majorbio BioTech Co., Ltd (China). A paired-end library with an average of a 450-bp insertion was constructed. After sequencing using the Illumina HiSeq 2000 platform (Illumina, USA) and quality control, clean reads were aligned to the DN13 strain genome using BWA (Burrows-Wheeler Aligner) software (Li and Durbin, 2009). GATK (the Genome Analysis Toolkit) software (https://software.broadinstitute.org/gatk/) was used to re-align reads near putative insertions or deletions (InDels) to eliminate false positive single nucleotide polymorphisms (SNPs). VarScan (Koboldt et al., 2009) was used to detect SNPs and small InDels, which were further filtered to obtain high confidence data.

### Analysis of Nucleotide Variations in the Mouse-Adapted Strains

**Figure 1:**
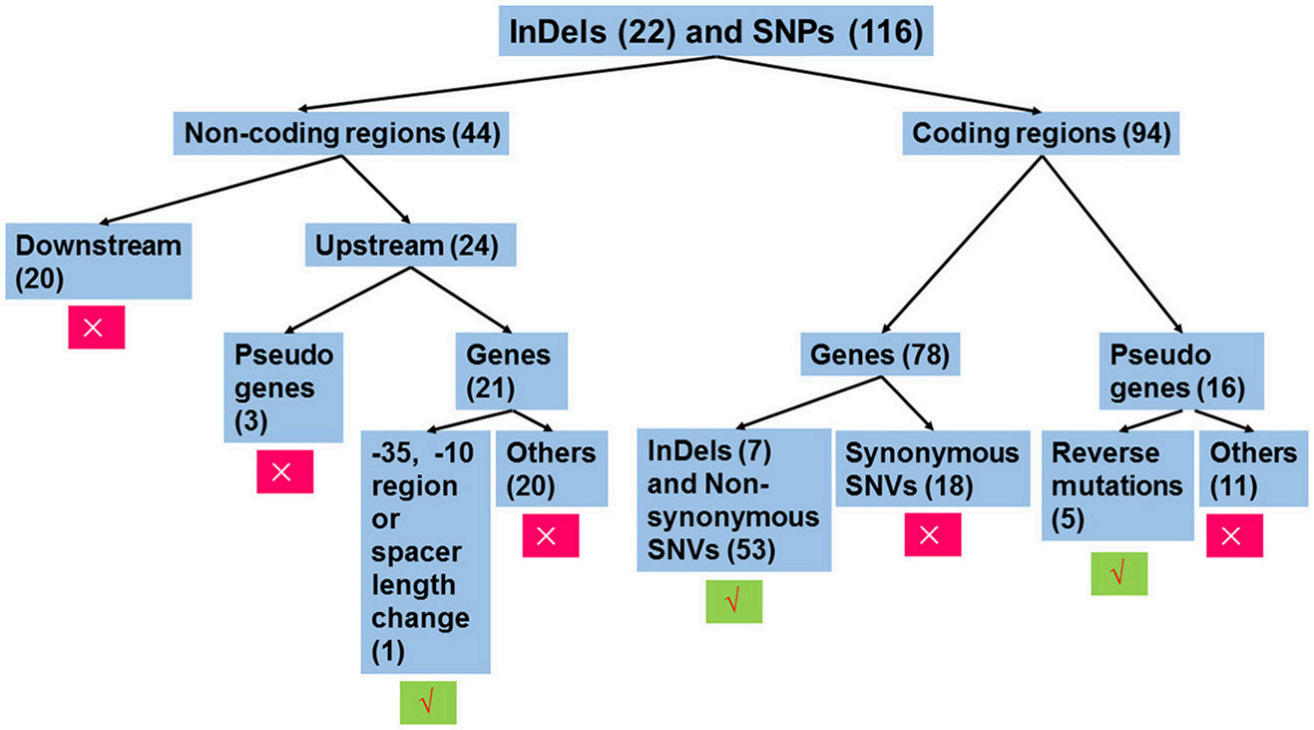
Workflow of sequence analysis to predict the effect of mutations on gene functions. Variations that affect the transcription of gene(s) or amino sequences were thought to be functional mutations. Otherwise, these were treated as silent mutations. Insertions or deletions (InDels) and single nucleotide polymorphisms (SNPs) in non-coding regions and coding regions were separately treated. For mutations in non-coding regions, only those mutations that changed the −35 hexamer, −10 hexamer, or their spacing length were further analyzed, since these mutations potentially affect promoter strength. For variations in coding regions, mutations leading to changes in amino acid sequences were analyzed, including functional ORFs with InDels or non-synonymous substitutions and pseudo genes with function restoration. The number in the parenthesis indicates the number of mutations belonging to the group. Symbols × and √ indicate exclusion and inclusion for further analysis, respectively.

The workflow of sequence analysis is illustrated in Figure 1. For variations in non-coding regions, the effect on promoter activity was mainly considered. In contrast, variations located down-stream of two ORFs (open reading frames) in opposite directions and mutations up-stream of pseudo genes were excluded from further analysis. Promoters were predicted using the online tool, BPROM (Li and Durbin, 2009). For mutations in the coding region, mutations in pseudo genes were thought to be silent, except for those leading to restoration of functional ORFs. In addition, synonymous replacements in functional ORFs were also treated as silent mutations. Based on these rules, mutations were analyzed individually.

### PCR Amplification and Sequencing

High fidelity polymerase (Takara, China) was used for PCR amplification. Primers used in this study are listed in Table 2 and Table S1. Product sequences were determined by Sanger sequencing at BIOSUNE Biotechnology Co., Ltd (China).

**Table 2:** Primers used in this study.

**Primer sequences (5′-3′)**		**Description**
A1	aaaaCTGCAGggagatacgaccattaattcc	For amplification of A6M16_09815
A2	taaatcaattttattaaagttcatataatcctccaaaaaattgtgtac	promoter from DN13 or SS9-P10
A3	atgaactttaataaaattgatttaga	For amplification of
A4	gcgGGATCCtaattcgatgggttccgagg	acetyltransferase-encoding sequence and its terminator from pSET1
P1	gcgGGATCCcagctatcacctatcttcgc	For amplification of downstream homologous
P2	cgGAATTCtatcgttcctaccaaaagcac	sequences of *srtR*
P3	cgcGTCGACatgaactttaataaaattgatttaga	For amplification of chloramphenicol
P4	gcgGGATCCttataaaagccagtcattaggc	acetyltransferase coding sequence
P5	cccAAGCTTctggatgccattgccattgt	For amplification of upstream homologous
P6	cgcGTCGACcatttctttctcctttcaggaa	sequences of *srtR*
P7	gcgGGATCCagtttgcaagccagtcttgc	For amplification of promoter and coding
P8	cgGAATTCagcaaaaccgcttctggcat	region of *srtR*
U1	cgagctcaaccaagccatgc	For detection of *srtR* in *S. suis*
U2	tcaaagctagacttgtggtagtt	
U3	atgaactttggacagcaaatca	For detection of *srtR* in *S. suis*
U4	ttatttatgttcttttctaaagcg	

### CAT Assay

Promoter strength was compared by fusing promoter sequences to a chloramphenicol acetyltransferase-encoding sequence and subsequently determining the maximum chloramphenicol concentration that bacteria are able to resist. Primer A1 and A2 were used to amplify promoter of A6M16_09815 from DN13 and SS9-P10, respectively. Primers A3 and A4 were used to amplify acetyltransferase-encoding sequence and its terminator from pSET1. Promoter originated from DN13 or SS9-P10 were fused with acetyltransferase-encoding gene, respectively, by overlap PCR as described (Heckman and Pease, 2007). Subsequently, the fused DNA segments were cloned into pSET2 between PstI and BamHI and designated as pSET2-PF0 (promoter of acetyltransferase-encoding gene originated from DN13) and pSET2-PF10 (promoter of acetyltransferase-encoding gene originated from SS9-P10). After introducing the recombinant plasmids into DN13 by electroporation, positive transformant containing pSET2-PF0 or pSET2-PF10, were streaked onto BHI agar with chloramphenicol ranging from 8 to 128 μg/ml at 2-fold increases to determine the maximum chloramphenicol concentration that the transformants are tolerant.

### Methyl Methane Sulfonate Sensitivity Assay

To compare the DNA ligase activity between DN13 and SS9-P10, a DNA damaging agent, methyl methane sulfonate, was used to treat the cells and the viabilities were recorded as previously described with minor modifications (Sriskanda et al., 1999). Briefly, treatment of bacteria was carried out as those described in “Oxidative stress,” except that 0.1 M H_2_O_2_ was replaced by 0.1% methyl methane sulfonate.

### Construction of Non-polar Knock-Out Mutant and Complement Strains

The isogenic mutant was generated by a homologous recombination-based method as previously described (Takamatsu et al., 2001b). The recombinant plasmid was constructed by double digestion of the vector with endonucleases, which was followed by ligation with identically-digested PCR products. Primers used in this study are listed in Table 2. In detail, the down-stream homologous segment amplified by primers P1 and P2, the chloramphenicol acetyltransferase-coding sequence amplified by primers P3 and P4, and the up-stream homologous segment amplified by primers P5 and P6 were step-wise inserted into the *S. suis* thermosensitive suicide vector pSET4s at BamHI/EcoRI, EcoRI/SalI, SalI/HindIII sites, respectively, to construct the knock-out vector pSET4s-srtR. The preparation of competent streptococcal cells and electroporation conditions were conducted according to a previously described method (Takamatsu et al., 2001a). Briefly, overnight *S. suis* cultures were inoculated into fresh BHI containing 40 mM DL-Threonine for approximately 3 h at 37°C with shaking (OD_600_ = 0.3–0.4), and cells were harvested by centrifugation at 1,000 × *g* at 4°C. The pellets were subsequently washed with ice-cold chemical transformation buffer [55 mM MnCl_2_, 15 mM CaCl_2_, 250 mM KCl, and 10 mM Pipes (piperazine-N,N′-bis(2- ethanesulfonic acid), pH. 6.7)] and suspended in the buffer. After a 30-min incubation on ice, bacteria were washed twice with electroporation buffer (0.3 M sucrose, 2 mM potassium phosphate, pH 8.4) and were suspended with electroporation buffer containing 15% glycerol for transformation. Plasmid or ligation mixture was added to 100 μl of competent cells and was transferred to a 2-mm gap electroporation cuvette and pulsed immediately with a Multiporator (Eppendorf, Germany) at 2,500 V. BHI was added to the cuvette and then transferred to sterile 1.5-ml Eppendorf tube. After a 2-h incubation, cells were centrifuged and spread onto BHI agar with appropriate antibiotics. After introducing pSET4s-srtR into SS9-P10, double-crossover *srtR* deletion mutants (SS9-P10:ΔsrtR) were screened. The DNA fragment containing the *srtR* coding region and its promoter were amplified by PCR using primers P7 and P8. The resultant product was then inserted into pSET2 at BamHI/EcoRI sites to generate pSET2-srtR. This plasmid was used to introduce a functional *srtR* gene into DN13, DN13:ΔsrtR, and SS9-P10:ΔsrtR to generate complementing strains DN13-srtR, DN13:CΔsrtR, and SS9-P10:CΔsrtR, respectively. Empty pSET2 was introduced into DN13 as a control to exclude the effect of the vector.

### Virulence Assays

To determine the role of SrtR in virulence in a mouse model, 9 × 10^5^ CFU of SS9-P10, SS9-P10:ΔsrtR, and SS9-P10:CΔsrtR were intraperitoneally administrated into groups of mice (*n* = 8). In contrast, 10^7^ CFU of DN13, DN13:ΔsrtR, and DN13:CΔsrtR were used for challenge for another three groups of mice. Other experimental details were the same as those described in the “Determination of the 50% lethal dose (LD_50_) of mouse-adapted strains” section.

### Statistical Analysis

GraphPad Prism version 6.0 (Graphpad software, USA) was used for statistical analysis. One-way analysis of variance (ANOVA) with Kruskal-Wallis test was used to analyze biofilm formation and for thermal and oxidative stress assays. Errors were expressed as mean ± standard deviation unless otherwise other mentioned. The comparison of survival rates among related groups was analyzed with the Log-rank (Mantel-Cox) test. *P* values less than 0.05 and 0.01, were considered significant and highly significant, respectively.

### Accession Numbers

The whole genome sequence of the parental strain DN13 was deposited in GenBank (https://www.ncbi.nlm.nih.gov/nuccore/) under accession number CP015557. Original whole genome resequencing data of SS9-P10, SS9-P20, and SS9-P30 were deposited in the Sequence Data Archive (https://www.ncbi.nlm.nih.gov/sra/) under accession numbers SRR6233341, SRR6233342, and SRR6233343, respectively.

## Results

### Serial Passage of SS9 in Mice Results in Increased Virulence

The DN13 strain used for mouse passage experiments was recovered from a diseased pig with bacteremia. Three genes extensively used for the prediction of virulence, including *sly, mrp*, and *efp* (Fittipaldi et al., 2012; Dong et al., 2017), were found to be absent in the DN13 strain, as detected by PCR, described elsewhere (Silva et al., 2006), and further confirmed by whole genome analysis. Further multi-locus genotyping using house-keeping genes *cpn60, dpr, recA, aroA, thrA, gki*, and *mutS* (King et al., 2002) identified this strain as sequence type 243 (ST243). SS9, belonging to ST243, was also isolated from diseased pigs in other areas in China (Dong et al., 2017; Lai et al., 2017).

Two groups of mice (*n* = 3) were challenged with approximately 10^7^ CFUs of passage 10 strains via tail vein and intraperitoneal injection, respectively. All three intraperitoneally-challenged mice died, whereas two tail vein-injected mice survived, indicating that the route of SS9 administration might affect virulence, as reported previously (Beineke et al., 2008). To screen for highly-virulent strains, intraperitoneal injection was performed for the following challenges and the dose of bacteria for passage in mice was decreased to 10^6^ CFU/mouse. For the following 20 passages, most infected mice died after challenge. To our surprise, the LD_50_ values of SS9-P10, SS9-P20, and SS9-P30 were similar, ranging from 3.2–7.1 × 10^4^ CFUs (Table 3), indicating an increase in virulence of at least 140-fold after 10 or more passages in mice.

**Table 3:** LD_50_ of the parental strain and the adapted strains.

**Bacterial strain**	**LD_**50**_**
DN13^a^	>10^7^ CFU
SS9-P10	5.3 × 10^4^ CFU
SS9-P20	7.1 × 10^4^ CFU
SS9-P30	3.2 × 10^4^ CFU

a *Before passaging, a group of mice (n = 5) were challenged with 10^7^ CFU/mouse and no death was observed and therefore LD_50_ was viewed as high than 10^7^ CFU*.

### Stress Tolerance and Biofilm Formation Are Enhanced in the Adapted Strains

**Figure 2:**
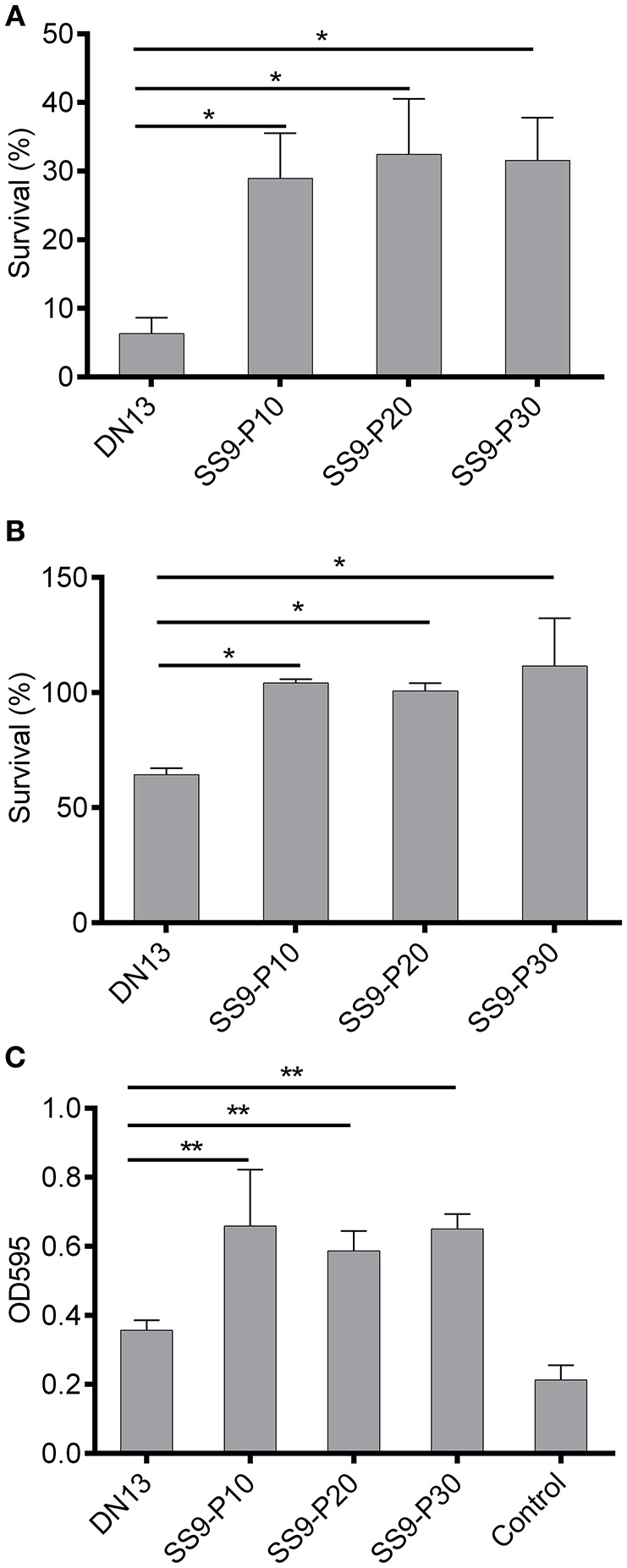
Increased tolerance to oxidative and high-temperature stress and biofilm formation in mouse-adapted *Streptococcus suis* strains. Exposure of late-log phage *S. suis* to 0.1 M H_2_O_2_ for 20 min was used to evaluate sensitivity to oxidative stress and treatment with sterile PBS was used as the control. The survival percentage was calculated based on the cell number in the H_2_O_2_-treatment group divided by the cell number in the control group. Late log-phase *S. suis* were plated onto BHI agar after six 10-fold dilutions, and growth at 42°C (37°C as the control) was used to induce high-temperature stress. The survival percentage was calculated as with the H_2_O_2_ challenge assay. Biofilms were induced by static culture of overnight cultures of *S. suis* for 3 days. After discarding planktonic bacteria and washing with sterile PBS twice, the biofilm was stained with crystal violet and then biofilm-absorbed dyes were dissolved in acetic acid for readings at OD_595_ (see materials and methods). In this part, experiments were carried out in duplicate and repeated three times. Values were expressed as mean percentages with standard deviation. One-way analysis of variance (ANOVA) with Kruskal-Wallis test was used to analyze biofilm formation and for oxidative and thermal stress assays. Oxidative stress **(A)**, high temperature stress **(B)**, capacity for biofilm formation **(C)**. *P < 0.05, **P < 0.01.

A series of virulence-associated phenotypes were compared between the parental strain and the adapted strains. The results of oxidant challenge showed that only approximately 6% of the DN13 strain survived after a 20-min exposure to 0.1 M H_2_O_2_, whereas at least 24% of the adapted strains remained viable (Figure 2A). For temperature stress, ~63% of the DN13 bacteria were viable at 42°C, whereas incubation at 42°C did not result in significant death for the passaged strains. (Figure 2B). These data showed that resistance to H_2_O_2_ and high temperature are significantly enhanced (*P* < 0.01) in the mouse-adapted strains. Similarly, the capacity for biofilm formation was also significantly increased (*P* < 0.05) in the adapted strains (Figure 2C).

### Whole Genome Analysis Identifies an XRE Family Transcriptional Regulator That Might Be Involved in Stress Tolerance

To identify genes that are involved in stress tolerance, the parental strain and the adapted strains were subjected to whole genome sequencing and whole genome resequencing, respectively. Compared to the parental strain, 150 (Tables S2, S3), 155 (Tables S4, S5), and 153 (Tables S6, S7) mutations were detected in the SS9-P10, SS9-P20, SS9-P30 strains, respectively, and 138 of these were commonly identified among the three strains (Table S8). Considering that the phenotypes of these three adapted strains were highly similar, only mutations that commonly existed among these strains were analyzed. To validate the high-throughput sequence data, 10 of 138 mutation sites in the SS9-P10 and their counterpart in the DN13 were sequenced by the Sanger method after PCR amplification (primers listed in Table S1). The results of Sanger sequencing were consistent with the whole genome sequencing and resequencing data.

As illustrated in Figure 1, we individually analyzed mutations in non-coding regions (Table S9) and coding regions (Table S10). For the former, conserved elements of the promoter including the −35 hexamer (TTGACA), the −10 hexamer (TATAAT), and their spacing length (mostly 17 ± 1 bp, but can range from 15 to 21 bp) (Shainheit et al., 2014; Takeuchi et al., 2014; Liao et al., 2016) that were similar to those in *E. coli* (Mitchell et al., 2003) were taken into account for changes in promoter activity.

As a consequence, 20 of 21 SNPs or InDels in non-coding regions were thought to be silent, as these variations did not affect −35 or −10 regions or their spacing length (Data Sheet 1). A “TATA” deletion up-stream of a hypothetical protein (A6M16_09815), resulted in homology changes to the −10 hexamer consensus (TATATA to TATAAT), potentially contributing to increased promoter activity (Figure S1). Consistent with this prediction, the mutated promoter-driven chloramphenicol acetyltransferase showed 2-fold resistance to chloramphenicol compared to that of DN13 (64 μg/ml vs. 32 μg/ml).

In coding-region, 65 mutations lead to changes in the amino acid sequence of 60 proteins, including premature stop, C-terminus extension caused by a stop codon mutation, C-terminus replacement resulting from frameshifts, deletions in the inner part as a consequence of codon deletions, and single amino acid replacements resulting from single nucleotide substitutions.

In *S. suis*, at least 100 genes were reported to be associated with virulence, supported by virulence evaluation of single gene-deficient mutants in animals. Products of these genes are involved in a wide range of biological pathways including capsule biosynthesis, regulation, metabolic pathway, with potential roles in pathogenesis by helping the bacteria to resist to the host defense system and adhere to host cells et al. The virulence of these deficient strains declines or is maintained after a single gene deletion (Segura et al., 2017), with the exception *CovR* (Pan et al., 2009), a negative regulator of virulence. For this reason, loss-of-function mutations have limited potential to increase virulence, even though loss of metabolic function can cause virulence increase in some bacterial species, including *Shigella spp, Yersinia pestis, E. coli*, and *Streptococcus agalactiae* (Domelier et al., 2006). Additionally, it is difficult to predict the effects of single amino acid replacements on the function of bacterial proteins (Olsen et al., 2010). Therefore, we focused on genes with multiple changes in amino acids, especially gain-of function mutations.

Stop codon mutations were identified in four putative pseudo genes, encoding two hypothetical proteins, an XRE family transcriptional regulator, and a LacI family transcriptional regulator, leading to carboxyl extension of these peptides in SS9-P10 (Table 4, Data Sheet 2). Further BLASTp analysis suggested that the resulting peptides were highly homologous to functional proteins, suggesting the occurrence of reverse mutations (Data Sheet 2). Another two genes also annotated as pseudo genes, encoding Ide_Suis_ and a prepilin peptidase, with InDels, were determined to lead to frameshifts at the carboxyl terminus in SS9-P10, and were also predicted to be reverse mutations (Table 4, Data Sheet 2). Among these variations, a repeat 12-codon deletion in the gene encoding a NAD^+^-dependent DNA ligase in SS9-P10 was also predicted to be a reverse mutation.

**Table 4:** Genes associated with reverse mutation in the mouse-adapted strains.

**Location**	**Locus tag**	**Variations**	**Description**	**Changes in product**
475215	A6M16_02490	A→G	DUF1827 family protein	. ^17^ →IIAEV^102^.
761229	ligA	“GGACCAACCTAGCGTATCAGATGC AGAATATGATAC” deletion	DNA ligase (NAD(+)) LigA	^23^DQPSVSDAEYDTDQPSVSDAEYDT→ ^46^DQPSVSDAEYDT
953178	A6M16_04800	T→C	Hypothetical protein	.^105^ →HSLSQSFRDKYMR.^120^
1334824	A6M16_06565	“A” insertion	Ide*_*Ssuis*_*	^1090^LFLVVL^1095^.→ ^1090^FVLGSTLGLLKKRRK.
1589362	A6M16_07840	“AT” deletion	Prepilin peptidase	^169^YVDLQNSSGLSRLVPS^184^.→^169^CGF TELLWIIQISSLLGLLVF TIFKPKSIPYVPLLFLSSIPIILCI.
1399963	A6M16_06855	A→G	XRE family transcriptional regulator	EGRRT.^86^→RKEHK.^159^
2096887	A6M16_10240	T→G	LacI family transcriptional regulator	.^228^→TITEL.^328^

*ligA* encodes an NAD-dependent DNA ligase that is responsible for DNA repair, recombination, and replication. In the mouse-adapted strains, 12 amino acids were deleted, resulting in a reverse mutation (Data Sheet 2). However, these amino acids are not located in well-known functional domains (Doherty and Suh, 2000), consistent with the result indicating no significant differences regarding sensitivity to the DNA-damaging agent methyl methane sulfonate (Figure S2).

*ide*_*Ssuis*_ encodes an immunoglobulin M-degrading enzyme in *S. suis* that selectively degrades immunoglobulin M of swine, but not mice, leading to complement evasion during the early stage of immune defense, which is dominated by immunoglobulin M (Seele et al., 2013, 2015). Therefore, the role of Ide_Ssuis_ in mouse pathogenesis remains unknown, even though the function of this protein was potentially improved.

Prepilin peptidases cleave the leader sequences from pilin, prepilin-like proteins, or pseudopilin before secretion via the type II protein secretion system in Gram-negative bacteria and the type IV pilus system of Gram-positive and Gram-negative bacteria (Liles et al., 1999; Korotkov et al., 2012). A6M16_07840 in DN13 was found to share homology with prepilin peptidases from *Pseudomonas aeruginosa, Vibrio cholera, Neisseria gonorrhoeae, Klebsiella oxytoca*, and *Legionella pneumophila* (Zhang et al., 1994; Liles et al., 1999), especially with respect to the amino-terminal conserved dithiol motif. In *Legionella pneumophila*, the deletion of prepilin peptidase was reported to affect the secretion of proteins, thereby impairing bacterial survival and virulence in the host (Liles et al., 1999). Reverse mutations in the prepilin peptidase in the mouse-adapted strains might have a positive effect on the cleavage of leader sequences before protein secretion, thereby facilitating pathogenesis.

Another gene, a LacI family transcriptional regulator, exhibited homology to the LacI-GalR family repressor, *malR*, which is involved in maltosaccharide utilization in *Streptococcus pneumoniae* (Puyet et al., 1993). This gene is responsible for the persistence of group A *Streptococcus* in the oropharynx, but not invasive infection (Shelburne et al., 2007, 2011). Like *malR, pulA* is another member of the MalR regulon, which encodes a cell-wall anchored carbohydrate-binding and degrading enzyme, contributing to virulence and pathogenesis via eukaryotic cell adhesion. In the adapted strains, *pulA* (A6M16_10245) was found to have a single amino acid replacement with an unknown effect on virulence (Table S10).

Even though these 4 reversion-associated genes might have positive effects on virulence in the adapted strains, the functions of these genes were not reported to be related to oxidative and thermal stress tolerance.

On the other hand, members of the XRE family transcriptional regulator might have roles in the stress response and virulence. For example, the XRE family transcriptional regulator, XdrA, in *Staphylococcus aureus* was found to regulate the expression of the virulence factor, *spa* (McCallum et al., 2010). In addition, expression of members of the XRE family of transcriptional regulators was reported to be up-regulated during high temperature stress in *Arthrospira platensis* (Panyakampol et al., 2015) and down-regulated in an H_2_O_2_-sensitive *Rhizobium etli* mutant (Martínez-Salazar et al., 2009).

SrtR contains a helix-turn-helix domain with a non-specific DNA binding site at the amino end and an uncharacterized DUF3955 domain at the carboxyl terminus. However, SrtR contains a premature stop after the helix-turn-helix domain in DN13 whereas full length of SrtR was predicted in SS9-P10. BLASTp analysis showed that 25 of 35 (71.4%) *S. suis* genomes harbor this gene and 24 of them encode functional SrtR, except for DN13. Further, SrtR is conserved among these genomes, specifically with 82% identity among the strains. Consistent with this, 64 of 85 (75.3%) clinical *S. suis* serotype 2 (45/65) and 9 (19/20) strains were found to contain functional *srtR* and SrtR sequences, which showed more than 84% identity among the strains (data not shown), suggesting that *srtR* might have an important function in this species. SrtR was found to be widely distributed in a wide range of facultative anaerobic or anaerobic Gram-positive bacteria, including many health-threatening pathogens such as *E. faecium* and *C. difficile* (Figure S3). This led us to hypothesize that *srtR* participates in stress tolerance.

### Knock-Out and Complement Strains Confirm That SrtR Contributes to Stress Response and Virulence but Not the Ability to Biofilm Formation

**Figure 3:**
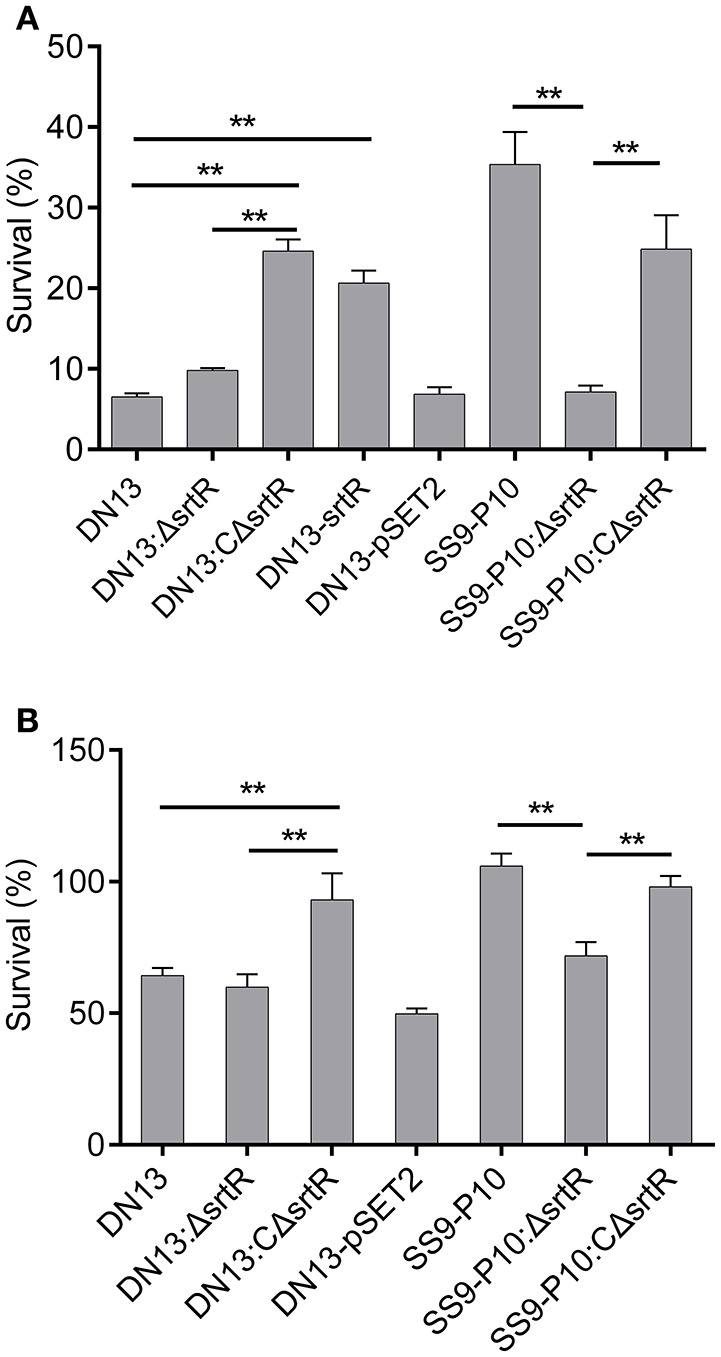
SrtR contributes to oxidant and high-temperature tolerance in *Streptococcus suis*. The role of *srtR* was evaluated by comparing the same background strains (DN13, DN13:ΔsrtR vs. DN13:CΔsrtR and DN13- srtR, SS9-P10:ΔsrtR vs. SS9-P10 as well as SS9-P10:CΔsrtR) with or without functional *srtR*. All experiments in this part were conducted in duplicate and repeated three times. Values were expressed as mean percentages with standard deviation. One-way analysis of variance (ANOVA) with Kruskal-Wallis test was used to assess oxidative and thermal stress assays. Plasmid-encoded *srtR* significantly increased resistance to H_2_O_2_ challenge in SrtR-deficient strains; DN13, DN13:ΔsrtR, and SS9-P10-ΔsrtR **(A)**. Growth at 42°C was significantly improved by the introduction of plasmid-encoded *srtR* into SrtR-deficient strains; DN13:ΔsrtR, and SS9-P10-ΔsrtR **(B)**. Survival data of DN13 and SS9-P10 at 42°C were identical to those displayed in Figure 2B, as thermal tolerance assays with DN13, the mouse-adapted strains, and engineered strains were conducted at the same time. **P < 0.01.

**Figure 4:**
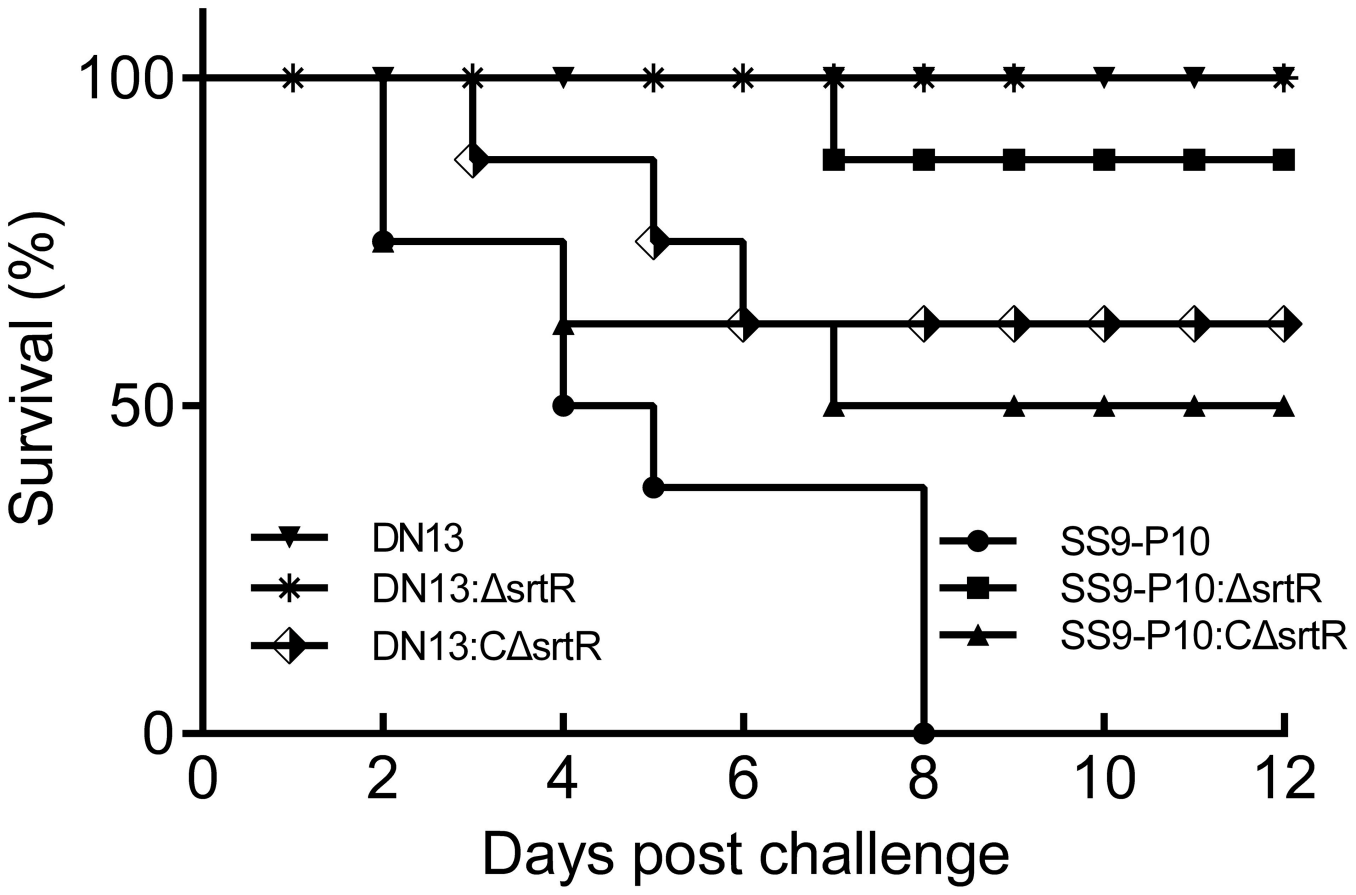
*Streptococcus suis* SrtR is a virulence factor. Using a mouse model of infection, 10^7^ colony forming units (CFUs) of DN13, DN13:ΔsrtR and DN13:CΔsrtR, and 9 × 10^5^ CFUs of SS9-10, SS9-P10:ΔsrtR, and SS9-P10:CΔsrtR in 100 μl of sterile PBS were used to challenge a group of animals (*n* = 8) via intraperitoneal injection. Survival was observed daily for 12 days. Comparison of survival rates was analyzed with the Log-rank (Mantel-Cox) test. The SrtR-expressed strains were virulent than their SrtR-deficient counterpart strains (DN13, DN13: ΔsrtR vs. DN13:C ΔsrtR, *P* < 0.01. SS9-10, SS9-P10:CΔsrtR vs. S9-P10:ΔsrtR, *P* = 0.07).

To test the hypothesis that *srtR* has a role in stress tolerance, we first introduced a plasmid-borne *srtR* into DN13 (DN13-srtR) and resistance of this complementing strain to H_2_O_2_ was tested. The results showed that DN13-srtR showed reduced sensitivity to H_2_O_2_ challenge. To further investigate its role in stress tolerance and virulence in mice, we constructed DN13 and SS9-P10-background deletion mutants and complementing strains. As expected, DN13:ΔsrtR and SS9-P10:ΔsrtR were sensitive to H_2_O_2_ challenge, compared to SS9-P10, exhibiting less than 10% survival. In contrast, resistance was significantly increased in both DN13:CΔsrtR and SS9-P10:CΔsrtR (Figure 3A). The growth of the *srtR* deletion mutants was impaired at 42°C but were restored in the mutants harboring the plasmid-encoded *srtR* (Figure 3B). In addition, the role of *srtR* in virulence was validated using a mouse infection model. After challenge with a 17LD_50_ (9 × 10^5^) dose of SS9-P10, no mice survived in this group. In contrast, seven of eight mice survived in the SS9-P10:ΔsrtR group and four of eight mice survived in the SS9-P10:CΔsrtR group after challenge with a similar dose (Figure 4). Consistent with the previous evaluation of virulence, no mice died in the DN13 group after a challenge with 10^7^ CFU/mouse. Similar to DN13, DN13:ΔsrtR was avirulent. In contrast, three of eight mice in the DN13:CΔsrtR were dead. In summary, these data suggest that *srtR* regulates stress tolerance and virulence in *S. suis*. However, SrtR is not involved in biofilm formation in *S. suis* (Figure S4).

## Discussion

To understand the mechanism of host adaptation and consequently the pathogenesis of SS9, the DN13 strain (ST243), recovered from a diseased pig, was selected for serial passage in mice. After 10 passages via intravenous administration, virulence was remarkably increased via intraperitoneal injection (> 140-fold), as evaluated by LD_50_ values, which appeared to be stable for the following 20 passages via intraperitoneal injection (Table 3), indicating genetic mutation occurred. A series of virulence-associated phenotypes were then compared between the parental strains and the mouse-adapted strains. For part, there were no differences in phenotypes including growth rate (Figure S5), resistance to mouse whole blood (Figure S6), and antibiotic resistance to aminoglycoside (Table S11), quinolone, and β-lactam families (Table S12). In contrast, tolerance to hydrogen peroxide and high temperature, as well as biofilm formation (Figure 2), were significantly increased.

After genome-wide analysis, 60 ORFs with potential functional variations and increase of promoter activity of a hypothetical protein were identified. However, oxidative stress response-related genes in Streptococci were not mutated in SS9-P10 compared to DN13, such as genes encoding two component system (Bugrysheva et al., 2011; Zhu et al., 2014), regulators (Brenot et al., 2005; Kajfasz et al., 2010; Zhang et al., 2012; Zheng et al., 2014), chaperones (Jones et al., 2001; Wu et al., 2011), iron transporters (Janulczyk et al., 2003; Turner et al., 2015) and oxidant-catalyzing enzymes (Yamamoto et al., 2006; Tang et al., 2012; Zheng et al., 2017). Among them, functions of several regulators and oxidant-detoxifying enzymes were well-characterized in *S. suis*. For instance, PerR regulates expression of proteins, such as Dpr (dps-like peroxide resistance protein), which can bind iron and block Fenton reaction (Zhang et al., 2012), and Spx proteins regulate SodA transforming superoxide into hydrogen peroxide and NADH oxidase catalyzing O_2_ into H_2_O to prevent formation of reactive oxygen species upon exposure to oxidant (Zheng et al., 2014).

Since well-known oxidative stress tolerance-associated genes were not varied in SS9-P10 compared to DN13, we tried to investigate genes with multiple changes in amino acids, especially those with reverse mutation as describe in Results. A total of seven genes were identified as listed in Table 4. Among them, Liga, Ide_Ssuis_, prepilin peptidase, and MalR homolog are not associated with stress response. In contrast, evidences indicated that members of XRE family transcriptional regulator were involved in stress response (Martínez-Salazar et al., 2009; McCallum et al., 2010; Panyakampol et al., 2015).

The XRE family transcriptional regulator, SrtR, in DN13 only contains a DNA non-specific binding domain resulting from a premature stop whereas a full length SrtR containing a further DUF3955 domain was identified in SS9-P10. To test whether SrtR has a role in oxidative stress tolerance, we first introduced a plasmid-born *srtR* into DN13 to test its sensitivity to H_2_O_2_ challenge. As suspected, sensitivity of DN13-srtR to H_2_O_2_ was reduced. The role of SrtR in the oxidative and high temperature stress but not biofilm formation was further confirmed by characterizing DN13- and SS9-P10-background deletion and complementing strains. Increased virulence in the strains with functional SrtR may partly be attributed to tolerance to oxidant and high temperature, since reactive oxygen species and temperature shift are important defense mechanisms of the innate immune system during the infection (Fang, 2011; Evans et al., 2015). However, SS9-P10:ΔsrtR was virulent in mice to some extent compared to DN13, indicating that other genes with potential functional mutation(s) might also contribute to increased virulence. In reality, it is well accepted that multiple factors participate in the pathogenesis of *S. suis* (Segura et al., 2017).

Cysteine residues in regulators including Spx proteins (Kajfasz et al., 2010; Zheng et al., 2014), members of the MarR (Chen et al., 2006, 2009; Lan et al., 2010; Lebreton et al., 2012; Zhang et al., 2012) and LsyR (Reen et al., 2013) families are partly or completely responsible for sensing oxidants, resulting in conformational changes and signal transduction, leading to the transcriptional activation of virulence factors. However, only a cysteine at 105 was found in SrtR. The exact mechanisms related to oxidant sensing, the target genes of SrtR, as well as interplay with other regulators (e.g., PerR, SpxA1 and SpxA2) require further investigation.

Taken together, reverse mutations of pseudo genes occurred during the process of serial passage in mice. Of these, *srtR* was confirmed to regulate the oxidative and high temperature stress response and virulence in mice. However, whether the SrtR gain-of-function leads to an increase in virulence in pigs is not clear. Our data identified the first member of the XRE family of transcriptional regulators that is responsible for oxidant tolerance and virulence in a murine model, facilitating our understanding of the pathogenesis of *S. suis* and provides insight into the mechanisms of adaptation after serial passage.

## Author Contributions

XY and YH designed the whole experiment. RW conducted the serial passage of SS9 in mice and contributed to whole genome sequencing. YH and QH performed the remaining assays. XY and YH analyzed the data and prepared the manuscript. DZ, RL, MG, and QY helped to prepare materials and to revise the manuscript.

### Conflict of Interest Statement

The authors declare that the research was conducted in the absence of any commercial or financial relationships that could be construed as a potential conflict of interest.

## Footnotes

^1^http://www.bioinformatics.babraham.ac.uk/projects/fastqc/

## Abbreviations

SS9: streptococcus suis serotype 9
SS9-P10: single isolate from passage 10
SS9-P20: single isolate from passage 20
SS9-P30: single isolate from passage 30
SrtR: stress response transcriptional regulator
ORF: open reading frame
BHI: brain heart infusion
CFUs: colony forming units
PBS: phosphate-buffered saline
LD50: 50% lethal dose
SNPs: single nucleotide polymorphisms
InDels: insertions or deletions
ST243: sequence type 243.
