# Supplementary material for: The XRE Family Transcriptional Regulator SrtR in Streptococcus suis Is Involved in Oxidant Tolerance and Virulence
Source: Front Cell Infect Microbiol. 2019 Jan 10;8:452. doi: 10.3389/fcimb.2018.00452 (PMC6335249; doi:10.3389/fcimb.2018.00452)
Supplement: Supplementary file 1 [file Data_Sheet_1.docx]

Supplementary Material

The XRE family transcriptional regulator SrtR in *Streptococcus suis* is involved in oxidant tolerance and virulence

Yuli Hu^1^, Qian Hu^1^, Rong Wei^1^, Runcheng Li^1^, Dun Zhao^1^, Meng Ge^1^, Qing Yao^1^, Xinglong Yu^1*^

*** Correspondence:** Xinglong Yu, xlyu999@126.com

**Data sheet 1**

Ranges of non-coding regions were highlighted with red. Location of mutation sites and variations were indicated before the nucleotide sequence. In the sequence, the -35 and -10 region of the promoter were highlighted with yellow and green, respectively. The mutation in each sequence was indicated with uppercase in bold. Underline indicates ribosome-binding site. For the non-coding region located at the adjacent upstream of an ORF in opposite direction, reverse complement sequence was listed. For non-coding region in upstream of two ORFs, both sequence and reverse complement sequence were shown.

Ranges of non-coding region, 75960-76272. Mutation site, 76180. Variation, A→T substitution

gataagatgtaagagcgtttagcaaccaaggcaaagataggagaatttacgatgaagtttacttctaggaaaatctatcttctttgctaagcttgtagctcgaactcaattaaaacaaccccgatacaaagtttgcttgccgatttgaacacaggcgaaagcctgtgtgaaaaagacaaactttcctagaaaccaaagtttctacgtcaagttttcta**A**tttcactttggctttttagcgccctttgctttgtatcatgaattaactagcaagtgactagatcaaactactagtaagaggagaaacaacaa

146351-147141, 146412, G→A substitution

gcggtaagtcaggaatctatgattccgtagacgcacccccgcaaggctgaataggttggtc**G**caagcgtagcaaagtgaactgctattcagctactgcgtacgaaagatgaacgatgaaaagagttgggagaccaactcttttctagtacaaaggctagattttctgtgaacgtagtgagtttagaaaatcagcctaggtaaaggctaaaatgcaacggatttccgttgcattttgtgcatcgcatatagtttctacgaagctaagcgagttagaaagtctaataaggtgtacttccaggacttgtgacggcacggagtgcctagtgccatacctagtctttctgggaagtgtgacctagaaagactggctaggtgctgccttgactgggaaataggcggtgagtcagttcaacatatctgggagatatgttgaggttagaaatagaagaagcgaagcttctgtcaaaaatgattccgtagattgttactttgatttttcttggtgacaacttcaaatagtctactgggctatttgaaccccgcaaggctgaatcttggtcgcaactcccattcagttactgcacaaaaatataaatgtaaaaaagacgttgtttttttgcagcgtctatttgtttgtcttacttctgggttccttcttgcagctgatttaaagtggcagattattggtttttcgaaaagctggcgtgattttccagtcgagcttaggagaaaagaaaagctgggcaggtatttgctattctattttccgaaatcatgttagaatagaggt

224750-225173, 225159, G→C substitution

aaattcatactcttcgaaaatcaaaattatccgttgtcaacttaccttgatgaactccagttctatcttcggcttcgtttcctaggctacttttgattttcattgagtgtcagaaaatattcttgattttttcgtgaaattagattaaaataaaactagataaaactgaataaccggttgatattgggtagaagggaaactgactgttcaatggacggaactctggagagaccattttggcaccgaaggggcaaggtagtcctgcttggaaaagtagagctactgaaactctcaggtaaaaggacagagcgttgaaaaataggctttttctgtatttttcacgttgattctagaggttgaagtgttcagcctctttttgttttttccggcagctttatcggattagaaa**G**gcttaggaggaact

722992-723137, 723112 and 723114, G→C substitution and C→A substitution, respectively.

ttataggtcatatgggactttttgtatatactcaaaaaggctccataatctccacggtggttttacccactacagaaattataga**G**cCctattttcttagttattaaagat

728871-729129, 728917, CA→AG substitution

atgaaattcgtgggtgaagggggagcgtgaaagaactcgactagt**CA**aaaaagagttcgtcaacaagtctttatttctagttgttgagctgaaacagtctatccccagactgtttcactcccacccccgcatagttgattagatcagattcggagtatgaaatacgaattgctgaaatttgttttggaactcctatctccaatctttcacagtccactgaactgctaaagccgatcaatcactgcgctgagatgttgat

984712-984924, 984839, C→A substitution

acctctattgtaacatgatttgacaaattattctaaaaaatgtataatgctgaaaatatatcctttaactgagtccagagaggcgaagaagggaacgtgataaatagggcaaatactgccttctttt**C**gtgtaaccttgctagccagcaaggttttttctctatatcgactttcttgttttctgtgtcattgttatatagaataggagaaaag

984712-984924, 984939, C→A substitution. Reverse complement of this region was listed since the transcription of the adjacent downstream ORF is in the opposite of DN13 genome sequence.

cttttctcctattctatataacaatgacacagaaaacaagaaagtcgatatagagaaaaaaccttgctggctagcaaggttacac**G**aaaagaaggcagtatttgccctatttatcacgttcccttcttcgcctctctggactcagttaaaggatatattttcagcattatacattttttagaataatttgtcaaatcatgttacaatagaggt

1099170-1099783, 1099443, G insertion. Reverse complement of this region was listed since the transcription of the adjacent downstream ORF is in the opposite of DN13 genome sequence.

catttattaattaaataaaccgtggaataccgctaaaaaaaatgagattttaggggctttttcgtcatgttttacttgtatttttgtatggtttgtgatacaataatattatcaagaaaacgaaaaaaggtgcaggagcgctaactcctacaccaccgattaagacaaaatagcgctttgcttaatcagtcaaacagtattgtagcataaagcaaacaatcacgtcaagactggagcaaagaaaaaactttgtttcggtctttttttcgtgagtgaagactatctgttttcttaggtttttaaagggattttgagctttcccaaaaatttagacacaaaca**C**atgatacaaaacacttgcatttgcaggtgtttttgttataatgggagtgttgactatgcacatcctgtgcaaccgcacgaatgccgttgctctgcaaaaattaagctcaaacgtcgttgacttaacttggtgaacctaagttctacctgtgtctacgtcgcctagtctgaacttaatttttattgagcaagataaagtggttcgtcccacggtactaggcgagtcttcacatcaaattcgggcaagacccaaaaatcataggaggtgcatg

1156402-1156507, 1156457, A insertion. Reverse complement of this region was listed since the transcription of the adjacent downstream ORF is in the opposite of DN13 genome sequence.

aatataaagaattacctttctgaaaaaatggtccattggaccatttttttg**T**tttgaattttgatatactagacatagtatattcaacgaaagaatgagaatttgat

1233140-1233232, 1233204, TA→AT substitution. Reverse complement of this region was listed since the transcription of the adjacent downstream ORF is in the opposite of DN13 genome sequence.

aaaccgagatttctcggtttttttctt**TA**gctatcattctagtattttaggggaatgtgatataataaagtatatcacgtatgaggaggctct

1260105-1260374, 1260309, G→A substitution. Reverse complement of this region was listed since the transcription of the adjacent downstream ORF is in the opposite of DN13 genome sequence.

ctgcaggaaaagatgcagatacacgttatatgacagatgcagtctttgaaaaagctgttttaatg**C**agcaaacaaagaactctatctgatcgacgatgccagtcatctccaaacctactggaaagaaccttacgtcaatcaagaaagtgaaaaattagtggcatttttcggtcaatatctggcttagttttcaatcgggtaagattatggtagaataaggcgactttcatttttaggagatacccatgacaaaagaacgttattggcatta

1325623-1327204, 1325845, C→A substitution.

tattttttgtctaactttttggggtcagtacacgagctagtagcctaacataaaaaatttttcgctttgattatatcatagggagggagtttttgcattcttttgagttttcgtataaagaactcgaccaagataaacatactaagattagtagtgaggaaatctccgacgggagaaagtactcactactttttctttatgataaagtagaggtgtcttgtt**C**agtcgagaactctt

1473833-1474219, 1474193, A insertion

ttttgtcctcctgagcttattaaccagttaacaattaactagtatacattttatgtttgatttttgcaagaaaaaaaacaactgacaatgattgttcgtgaataaattttccattccacgacaaacgcatgaaaaatttgatttataaaccatattttcacgactgctgtcagtttttagtagaaaaaatgtaatataatagcattgtaaatgagggattatccttgttaaagtgcaggttttacaaaaaaatttaaaaaaatgcatgcaacaagaaaatagttacatgccttttcttcatacagatagcgtgtttccatccctacgaccattgacaaagcaaacaaactatagtatatt**A**aaaatagggaaatacaaggagaatatt

Reverse complement

aatattctccttgtatttccctatttta**T**atatactatagtttgtttgctttgtcaatggtcgtagggatggaaacacgctatctgtatgaagaaaaggcatgtaactattttcttgttgcatgcatttttttaaatttttttgtaaaacctgcactttaacaaggataatccctcatttacaatgctattatattacattttttctactaaaaactgacagcagtcgtgaaaatatggtttataaatcaaatttttcatgcgtttgtcgtggaatggaaaatttattcacgaacaatcattgtcagttgttttttttcttgcaaaaatcaaacataaaatgtatactagttaattgttaactggttaataagctcaggaggacaaaa

1549503-1550043, 1549953, A deletion.

ttgaatttggtaatatgccatgaatatacaagcgtacatcgctgtgagttgcataaaaatgagcaatccgacattttcatagatatccatcctttcctcctttacttcttcttccacttcacaatctgccatatcacaaacgacagagccaacaaaccagctaaccaatagaccattgctttctttggcaaatggtggtatacttcaaataagaggttggggctgctcttaatctttattttgtaagttccgcttgtgctcaagcacttgtttgtgccataagcgagcttctcttactaagcctagaatgataatcgggatcgctaagtcgttatcagctaggctttttagtatgtccaccatttgcttttcctcctgttttattcggtcatttccctgaccttgactatattataatccgttttaaaacggatatcaactataaaaacg**A**aaaaaaaacgaatttttttgtgttttcattttacaaaaacgatttaaaacggtattattatagatagaaaagtaatcggaggtattaaaa

1643813-1643969, 1643846 and 1643861, G→T substitution and G→A substitution, respectively.

ctgcggctacctttttcaccaagggatgggagcg**G**gaaagaactcgact**G**gtcaaaaacatactaagattagtagtgaggaaatcttcgacgggagaaagtactcactactttttctttatgataaagtagaggtgtcttgttaagtcgagaactctt

Reverse complement

aagagttctcgacttaacaagacacctctactttatcataaagaaaaagtagtgagtactttctcccgtcgaagatttcctcactactaatcttagtatgtttttgac**C**agtcgagttctttc**C**cgctcccatcccttggtgaaaaaggtagccgcag

646330-1646422, 1646361, C insertion. Reverse complement of this region was listed since the transcription of the adjacent downstream ORF is in the opposite of DN13 genome sequence.

aatgttagaaacagcagaaatgctgttttttttaccttgacaaatatggttggaggggggg**G**taaaatagagagagaattggtggaggataaaa

1792351-1792831, 1792478, C insertion. Reverse complement of this region was listed since the transcription of the adjacent downstream ORF is in the opposite of DN13 genome sequence.

gaatctttctaaacaagataagagccgtcaggctctttttttgatataatatagtggatatggttaattaaaattgtcagaaaagactattttatagattaacactctctgaaaatcatcattaccaagaaaagaggctgggctcaagccccgcaccacttctcaaagttagcgtcaacatctcagagcagtagtggttgattgggtttaacagtccagtggagtgaggctgggctcaagccccgtaccgcttctcaacgttagtgtcaacatctcagcgcagtggttgattggcagatttgttcgtgttttacactccaaatctgacctaatcaactgtgcggggggggg**G**aaacgaactcttctatggatggtcgagttctttccccactgccaaggttgagtcttatcggtgtatctagttagcttggtcagttaatgtggtttttgaagaggacaaaaatgaaaaggagcacagc

1889686-1889830, 1889767, C→T substitution.

atgtcacttcctttcgcttcctctttattataccctatcatgcttttctttggaacaaaagggacacattggacaatctgt**C**ccttccttttttcttttggataatgtaaaatgaaagcgcaaacaataacaaaggagttttctc

Reverse complement

gagaaaactcctttgttattgtttgcgctttcattttacattatccaaaagaaaaaaggaagg**G**acagattgtccaatgtgtcccttttgttccaaagaaaagcatgatagggtataataaagaggaagcgaaaggaagtgacat

1969520-1969589, 1969525, T→C substitution. Reverse complement of this region was listed since the transcription of the adjacent downstream ORF is in the opposite of DN13 genome sequence.

acggtatgtttattggcttttttgaattttgtggtataatagaagtaattttgaaagatggagt**A**gcagtt

2002190-2002304, 2002222, TATA deletion. Reverse complement of this region was listed since the transcription of the adjacent downstream ORF is in the opposite of DN13 genome sequence. This mutation was thought to strength the promoter and was validated by CAT assay.

gataatacatgaaaattaggaggtaagtcttctaatttttttgtttttctacttgacaacgcatatatatatatatata**TATA**atggtgttgtacacaattttttggaggattat

2037247-2037438, 2037387, C→A substitution

taattaaaacacactttctttttcagattttacaatagtatcatgaataacaatttccagccaatatattttttctatcagagacattaaaaattttaatcaagtatcggagcataaaaaaagacctactcggtcttgaac**C**gcaccccaaaagttagacagaaaaaatctaacatttggggtgctattttt

Reverse complement

aaaaatagcaccccaaatgttagattttttctgtctaacttttggggtgc**G**gttcaagaccgagtaggtctttttttatgctccgatacttgattaaaatttttaatgtctctgatagaaaaaatatattggctggaaattgttattcatgatactattgtaaaatctgaaaaagaaagtgtgttttaatta
