# Supplementary material for: The XRE Family Transcriptional Regulator SrtR in Streptococcus suis Is Involved in Oxidant Tolerance and Virulence
Source: Front Cell Infect Microbiol. 2019 Jan 10;8:452. doi: 10.3389/fcimb.2018.00452 (PMC6335249; doi:10.3389/fcimb.2018.00452)
Supplement: Supplementary file 2 [file Data_Sheet_2.docx]

Supplementary Material

The XRE family transcriptional regulator SrtR in *Streptococcus suis* is involved in oxidant tolerance and virulence

Yuli Hu^1^, Qian Hu^1^, Rong Wei^1^, Runcheng Li^1^, Dun Zhao^1^, Meng Ge^1^, Qing Yao^1^, Xinglong Yu^1*^

*** Correspondence:** Xinglong Yu, xlyu999@126.com

**Data sheet 2**

Products of seven reverse mutation-associated genes in the SS9-P10 were compared with those of DN13 and GenBank. Origin of each sequence was indicated in the sequence title. The differences in amino acid sequences among DN13, SS9-P10, and representative sequence in GenBank were highlighted with yellow.

DUF1827 family protein (A6M16_02490)

DN13

MKIINTTNSHPNLVQS.

SS9-P10

MKIINTTNSHPNLVQSQLANTDAFLVETYSAGNTDVIFTQAPRHYELLISNKYRAVQQNEIEQIRDFFLHRKIDGRTINKAAIQTIHTDRLIEMSIPIIAEV.

BLAST

MKIINTTNSHPNLVQSQLANTDAFLVETYSAGNTDVIFTQAPRHYELLISNKYRAVQQNEIEQIRDFFLHRKIDGRTINKAAIQTIHTDRLIEMSIPIIAEV.

NAD^+^-dependent DNA ligase (*ligA*)

DN13

MKTRISELVSVLSQYAKEYYQLDQPSVSDAEYDTDQPSVSDAEYDTLYRELVELETAHPELILPDSPTHRVGGKVLDGFEKYSHVYPLFSLQDAFSREELEAFDQRVRKEFPQATYICELKIDGLSISLTYEAGNLVVGATRGDGSVGENITENLKRVADIPLTLPEAVDITVRGECYMPKASFDRVNKQRQEAGEAEFANPRNAAAGTLRQLDTGVVAQRGLATFLYQEASPSEATSQSQVLEKLDALGFVTNHEYCLAESIDDTWDFIEKIAERRDDLPYEIDGVVIKVNDLAIQEELGFTVKAPRWAVAYKFPAEEKEAEILSVDWTVGRTGVVTPTANLSPVQLAGTTVSRATLHNVDYIAEKDIRIGDTVIVYKAGDIIPAVLKVVDKYRSEQEVMPIPSHCPSCQSDLQHYEDEVALRCINPICPSQLMSKLEHFASRDAMNIAGLGSSIVEKLFGAGLVHDVADIYKLSVEDLLTLEGFKEKSADKLYQAIQTSKSNSAERLLFGLGIRHVGSKASKILVEKFGDLETLAFADQEAIASLEGLGQVIAKSLTTFFASEGAQQLLAELKEAKVNLTYLGQVVDENAALSGMTVVLTGKLERMKRNEAKAKLEALGANVAGSVSKKTNLVVAGTDAGSKLTKAQELGIEIKDEAWLESL.

SS9-P10

MKTRISELVSVLSQYAKEYYQLDQPSVSDAEYDTLYRELVELETAHPELILPDSPTHRVGGKVLDGFEKYSHVYPLFSLQDAFSREELEAFDQRVRKEFPQATYICELKIDGLSISLTYEAGNLVVGATRGDGSVGENITENLKRVADIPLTLPEAVDITVRGECYMPKASFDRVNKQRQEAGEAEFANPRNAAAGTLRQLDTGVVAQRGLATFLYQEASPSEATSQSQVLEKLDALGFVTNHEYCLAESIDDTWDFIEKIAERRDDLPYEIDGVVIKVNDLAIQEELGFTVKAPRWAVAYKFPAEEKEAEILSVDWTVGRTGVVTPTANLSPVQLAGTTVSRATLHNVDYIAEKDIRIGDTVIVYKAGDIIPAVLKVVDKYRSEQEVMPIPSHCPSCQSDLQHYEDEVALRCINPICPSQLMSKLEHFASRDAMNIAGLGSSIVEKLFGAGLVHDVADIYKLSVEDLLTLEGFKEKSADKLYQAIQTSKSNSAERLLFGLGIRHVGSKASKILVEKFGDLETLAFADQEAIASLEGLGQVIAKSLTTFFASEGAQQLLAELKEAKVNLTYLGQVVDENAALSGMTVVLTGKLERMKRNEAKAKLEALGANVAGSVSKKTNLVVAGTDAGSKLTKAQELGIEIKDEAWLESL.

BLAST

MKTRISELVSVLSQYAKEYYQLDQPSVSDAEYDTLYRELVELETAHPELILPDSPTHRVGGKVLDGFEKYSHVYPLFSLQDAFSREELEAFDQRVRKEFPQATYICELKIDGLSISLTYEAGNLVVGATRGDGSVGENITENLKRVADIPLTLPEAVDITVRGECYMPKASFDRVNKQRQEAGEAEFANPRNAAAGTLRQLDTGVVAQRGLATFLYQEASPSEATSQSQVLEKLDALGFVTNHEYCLAESIDDTWDFIEKIAERRDDLPYEIDGVVIKVNDLAIQEELGFTVKAPRWAVAYKFPAEEKEAEILSVDWTVGRTGVVTPTANLSPVQLAGTTVSRATLHNVDYIAEKDIRIGDTVIVYKAGDIIPAVLKVVDKYRSEQEVMPIPSHCPSCQSDLQHYEDEVALRCINPICPSQLMSKLEHFASRDAMNIAGLGSSIVEKLFGAGLVHDVADIYKLSVEDLLTLEGFKEKSADKLYQAIQTSKSNSAERLLFGLGIRHVGSKASKILVEKFGDLETLAFADQEAIASLEGLGQVIAKSLTTFFASEGAQQLLAELKEAKVNLTYLGQVVDENAALSGMTVVLTGKLERMKRNEAKAKLEALGANVAGSVSKKTNLVVAGTDAGSKLTKAQELGIEIKDEAWLESL.

Note: mutation at 761229 in ligA, leads to a repeat 12-condon deletion as indicated with underline. However, this deletion seems to be silent since well-known domains were not affected and sensitivity of DN13 to a DNA-damaging agent (methyl methane sulfonate) has no difference compared with SS9-P10. Functional domains of LigA are highlighted with green in sequence in SS9-P10. ELKIDGL, domain I. VRGECY, domain III. DGVVI, domain IV. AVAYKFPAEE, domain V.

Hypothetical protein (A6M16_04800)

DN13

MKQDIRLVRKQFRMTRQEEKQIKEMMKDQQIESFSEFLRHNLFKTDDDDKSIEMWFSLWQSQKLEQVSRDLYEVLVIAKQNHQVTQEHVSILLTCVQELIVEVS.

SS9-P10

MKQDIRLVRKQFRMTRQEEKQIKEMMKDQQIESFSEFLRHNLFKTDDDDKSIEMWFSLWQSQKLEQVSRDLYEVLVIAKQNHQVTQEHVSILLTCVQELIVEVSQSHSLSQSFRDKYMR.

BLAST

MKQDIRLVRKQFRMTRQEEKQIKEMMKDQQIESFSEFLRHNLFKTDDDDKSIEMWFSLWQSQKLEQVSRDLYEVLVIAKQNHQVTQEHVSILLTCVQELIVEVSQSHSLSQSFRDKYMR.

The immunoglobulin M-degrading enzyme of *Streptococcus suis* (A6M16_06565)

DN13

MRIQERFSLRKSAVGLVSVSLLCAIYTSTVAADTVVTGVNEIIEESQVKDEVSIESEKNESLDGSNIEIVEEIADNIPSPVIAEGEIAVEMKVDRGTENVVSRNDTEVTTSEQNQIEVTETKEILNQTSYQTESGEQRQIIWAHGITPPAMEQSGGFVKEKYGDYLNYTAPFKAGKGYYDTNKSLNASFIDLNLCFAAVSSNMVHWWLEQNSSYVERYLKEKNGTVNVGENYAITDLRRYIDSFQDQQNSRVFDMFKTYYGYRTNGFVSDALVDLFINGYKPKAQGGVNLEDSQLVPDSRGGFFYDVFKEKKLTNRIFSGSYERFGEDVRTVLESKGLLGLTYRTLGYATHIVTVWGAEYDNQGKIKAVYITDSDDQQEQIGLKRMGITRDASGNPRLNNHVKNNSAGALLDYVHTIRLGQDLWEEYFNPLAKSKETASQTLADTKKALDLSIQGQSELPESMRLIYLEKLNNLYNQGILSIQKAESSEMLSGALENGLNSLKSLDFPISEVGNALAPDLPVGDRSTVSDVDSLSSQETSSTNLEVDTENADLIADGADQLHFPVEVQTTSSVEAEGDNVFEQEADTLPIIIENKDEFGSELSRNMQTSETDSLVVAVEEDVKNDEVAQVEELLESEKVENQSSELLSDTLIVEGANDKEEDRVEAVVSEQPDSIPHQNVEISSVELTNVETESVVTPVNDAATPHGSPTYIDNSVTESVATPLEKDSIQAGETEIAEPTSSESTSVEAETVVTPVNDVATPHGSPTYIDNSVTESVATPLEKDSIQAGETEIAEPTSSESTSVEAELVDNSEIHSATSSVTPRGSSAYADSSTTESVATPLEKDSIQTREIENAEQTLSEPTNAEAESVATPLEKDSIQAGETEIAEPTSSKSTNAEAASVDNSEIHADTSLTAVSSVNLDNPVIEPVAIPLIGSKRDTNAEVEVSSLSKREVRKPNTEGLISVQSKIIKKELLEATIAQSSNSNSTEIGMSYQNTVLLESNNTERQASKAEIVMEHKETELVETVSSASEPVVLVENISQTSNNTIESGKNMGVQSQAGAKQILGVEQSSKVSTPTSRQIMGVGLLTLFLVVL.

SS9-P10

MRIQERFSLRKSAVGLVSVSLLCAIYTSTVAADTVVTGVNEIIEESQVKDEVSIESEKNESLDGSNIEIVEEIADNIPSPVIAEGEIAVEMKVDRGTENVVSRNDTEVTTSEQNQIEVTETKEILNQTSYQTESGEQRQIIWAHGITPPAMEQSGGFVKEKYGDYLNYTAPFKAGKGYYDTNKSLNASFIDLNLCFAAVSSNMVHWWLEQNSSYVERYLKEKNGTVNVGENYAITDLRRYIDSFQDQQNSRVFDMFKTYYGYRTNGFVSDALVDLFINGYKPKAQGGVNLEDSQLVPDSRGGFFYDVFKEKKLTNRIFSGSYERFGEDVRTVLESKGLLGLTYRTLGYATHIVTVWGAEYDNQGKIKAVYITDSDDQQEQIGLKRMGITRDASGNPRLNNHVKNNSAGALLDYVHTIRLGQDLWEEYFNPLAKSKETASQTLADTKKALDLSIQGQSELPESMRLIYLEKLNNLYNQGILSIQKAESSEMLSGALENGLNSLKSLDFPISEVGNALAPDLPVGDRSTVSDVDSLSSQETSSTNLEVDTENADLIADGADQLHFPVEVQTTSSVEAEGDNVFEQEADTLPIIIENKDEFGSELSRNMQTSETDSLVVAVEEDVKNDEVAQVEELLESEKVENQSSELLSDTLIVEGANDKEEDRVEAVVSEQPDSIPHQNVEISSVELTNVETESVVTPVNDAATPHGSPTYIDNSVTESVATPLEKDSIQAGETEIAEPTSSESTSVEAETVVTPVNDVATPHGSPTYIDNSVTESVATPLEKDSIQAGETEIAEPTSSESTSVEAELVDNSEIHSATSSVTPRGSSAYADSSTTESVATPLEKDSIQTREIENAEQTLSEPTNAEAESVATPLEKDSIQAGETEIAEPTSSKSTNAEAASVDNSEIHADTSLTAVSSVNLDNPVIEPVAIPLIGSKRDTNAEVEVSSLSKREVRKPNTEGLISVQSKIIKKELLEATIAQSSNSNSTEIGMSYQNTVLLESNNTERQASKAEIVMEHKETELVETVSSASEPVVLVENISQTSNNTIESGKNMGVQSQAGAKQILGVEQSSKVSTPTSRQIMGVGLLTFVLGSTLGLLKKRRK.

MRIQERFSLRKSAVGLVSVSLLCAIYTSTVAADTVVTGVNEIIEESQVKDEVSIESEKNESLDGSNIEIVEEIADNIPSPVIAEGEIAVEMKVDRGTENVVSRNDTEVTTSEQNQIEVTETKEILNQTSYQTESGEQRQIIWAHGITPPAMEQSGGFVKEKYGDYLNYTAPFKAGKGYYDTNKSLNASFIDLNLCFAAVSSNMVHWWLEQNSSYVERYLKEKNGTVNVGENYAITDLRRYIDSFQDQQNSRVFDMFKTYYGYRTNGFVSDALVDLFINGYKPKAQGGVNLEDSQLVPDSRGGFFYDVFKEKKLTNRIFSGSYERFGEDVRTVLESKGLLGLTYRTLGYATHIVTVWGAEYDNQGKIKAVYITDSDDQQEQIGLKRMGITRDASGNPRLNNHVKNNSAGALLDYVHTIRLGQDLWEEYFNPLAKSKETASQTLADTKKALDLSIQGQSELPESMRLIYLEKLNNLYNQGILSIQKAESSEMLSGALENGLNSLKSLDFPISEVGNALAPDLPVGDRSTVSDVDSLSSQETSSTNLEVDTENADLIADGADQLHFPVEVQTTSSVEAEGDNVFEQEADTLPIIIENKDEFGSELSRNMQTSETDSLVVAVEEDVKNDEVAQVEELLESEKVENQSSELLSDTLIVEGANDKEEDRVEAVVSEQPDSIPHQNVEISSVELTNVETESVVTPVNDAATPHGSPTYIDNSVTESVATPLEKDSIQAGETEIAEPTSSESTSVEAETVVTPVNDVATPHGSPTYIDNSVTESVATPLEKDSIQAGETEIAEPTSSESTSVEAELVDNSEIHSATSSVTPRGSSAYADSSTTESVATPLEKDSIQTREIENAEQTLSEPTNAEAESVATPLEKDSIQAGETEIAEPTSSKSTNAEAASVDNSEIHADTSLTAVSSVNLDNPVIEPVAIPLIGSKRDTNAEVEVSSLSKREVRKPNTEGLISVQSKIIKKELLEATIAQSSNSNSTEIGMSYQNTVLLESNNTERQASKAEIVMEHKETELVETVSSASEPVVLVENISQTSNNTIESGKNMGVQSQAGAKQILGVEQSSKVSTPTSRQIMGVGLLTLFVLGSTLGLLKKRRK.

*SrtR* (A6M16_06855)

DN13

MNFGQQIKDLRKKESLTQEQFALKLNVTRQAVSNWENDKNLPDLELLILMSSVFSISLDHLILGGTDMNNMTEKLVKDGREGRRT.

SS9-P10

MNFGQQIKDLRKKESLTQEQFALKLNVTRQAVSNWENDKNLPDLELLILMSSVFSISLDHLILGGTDMNNMTEKLVKDGREGRRTQMHLTITIIGAFLMVLGLFCFFVKANSVEYIDAEGILHENFYLIPIGFLFLLVGGMTSLISAITYLRFRKEHK.

BLAST

MNFGQQIKDLRKKEGLTQEQFALKLNVTRQAVSNWENDKNLPDLELLILMSSVFSISLDHLILGGTDMNNMTEKLVKDGREGRRTQMHLTITIIGSFLMLLGFVCFIIEANSVEYIDAEGILHENFYLIPVGYLLVFTGAIATLLSGLALHRFRKEYK.

Note: mutation at 1399963, contributes to C-terminal extension to a functional protein with helix-turn-helix domain and a DUF3955 domain, compared with that of DN13, which only contains the helix-turn-helix domain.

prepilin peptidase (A6M16_07840)

DN13

MKTIILFFLGASIGSFLGLVIDRFPEQSIISPSSHCNACKRRLKAWDLIPILSQLSTKSKCRYCKAKIPYWYLGLEFLAGLVVLLCHFQVLNLTETILSLAGLVLTIYDIKHQEYPFAVWLVFTFTALVLSQLNWLFCGFLLLAYLTEKWQFNIGSGDFLYLASLALIYVDLQNSSGLSRLVPS.

SS9-P10

MKTIILFFLGASIGSFLGLVIDRFPEQSIISPSSHCNACKRRLKAWDLIPILSQLSTKSKCRYCKAKIPYWYLGLEFLAGLVVLLCHFQVLNLTETILSLAGLVLTIYDIKHQEYPFAVWLVFTFTALVLSQLNWLFCGFLLLAYLTEKWQFNIGSGDFLYLASLALICGFTELLWIIQISSLLGLLVFTIFKPKSIPYVPLLFLSSIPIILCI.

BLAST

MKTIILFFLGASIGSFLGLVIDRFPEQSIISPSSHCNACKRRLKAWDLIPILSQLSTKSKCRYCKAKIPYWYLGLEFLAGLVVLLCHFQVLNLTETILSLAGLVLTIYDIKHQEYPFAVWLVFTFTALVLSQLNWLFCGFLLLAYLTEKWQFNIGSGDFLYLASLALICGFTELLWIIQISSLLGLLVFTIFKPKSIPYVPLLFLSSIPIILCI.

LacI family transcriptional regulator (A6M16_10240)

DN13

MRATIKDVAKLAGVSPSTVTRVIQNSSAISQKTKDLVRKAMADLNYHPNLNARSLVSSYTQVIGLVLPDDSDVFYQNPFFPTALRGISQVAADHNYAIQISTGKNEEQRLEAISQMVYGKRVDGLIFLYSKPDDPLVQLAIQHKFPFLILGKADSPFISLVDNDNIQAGFEATNYFINKGYKNIAFVAGNKELVVSQDRYTGYKNALKSHNIPLDENKVKFVSGFLL.

SS9-P10

MRATIKDVAKLAGVSPSTVTRVIQNSSAISQKTKDLVRKAMADLNYHPNLNARSLVSSYTQVIGLVLPDDSDVFYQNPFFPTALRGISQVAADHNYAIQISTGKNEEQRLEAISQMVYGKRVDGLIFLYSKPDDPLVQLAIQHKFPFLILGKADSPFISLVDNDNIQAGFEATNYFINKGYKNIAFVAGNKELVVSQDRYTGYKNALKSHNIPLDENKVKFVSGFLLEDSAYKISKKLLKQDLDAIVTTDTSVAEGVVKYLNEVGVKLPIISFDSVKPKLDIEAYVDVHAIKLGRVAFNTLHQIINDNKEDKQVCYRRVIPHTITEL.

BLAST

MRATIKDVAKLAGVSPSTVTRVIQNSSAISQKTKDLVRKAMADLNYHPNLNARSLVSSYTQVIGLVLPDDSDVFYQNPFFPTALRGISQVAADHNYAIQISTGKNEEQRLEAISQMVYGKRVDGLIFLYSKPDDPLVQLAIQHKFPFLILGKADSPFISLVDNDNIQAGFEATNYFINKGYKNIAFVAGNKELVVSQDRYTGYKNALKSHNIPLDENKVKFVSGFLLEDSAYKISKKLLKQDLDAIVTTDTSVAEGVVKYLNEVGVKLPIISFDSVKPKLDIEAYVDVHAIKLGRVAFNTLHQIINDNKEDKQVCYRRVIPHTITEL.
