# Supplementary material for: The XRE Family Transcriptional Regulator SrtR in Streptococcus suis Is Involved in Oxidant Tolerance and Virulence
Source: Front Cell Infect Microbiol. 2019 Jan 10;8:452. doi: 10.3389/fcimb.2018.00452 (PMC6335249; doi:10.3389/fcimb.2018.00452)
Supplement: Supplementary file 3 [file Data_Sheet_3.docx]

Supplementary Material

The XRE family transcriptional regulator srtR in *Streptococcus suis* is involved in oxidant tolerance and virulence

Yuli Hu^1^, Qian Hu^1^, Rong Wei^1^, Runcheng Li^1^, Dun Zhao^1^, Meng Ge^1^, Qing Yao^1^, Xinglong Yu^1*^

*** Correspondence:** Xinglong Yu, xlyu999@126.com


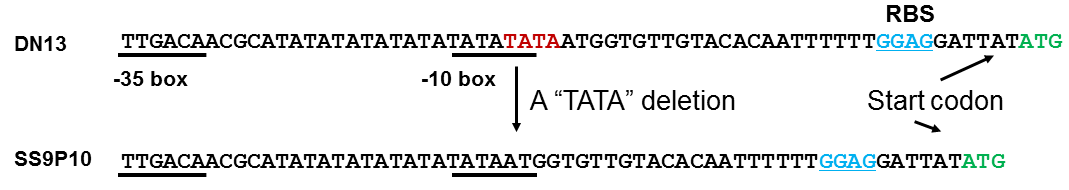


Figure S1. Changes to the −10 hexamer potentially affect promoter activity. “TTGACA” and “TATAAT” are most frequently found in the −35 and −10 regions of the promoter, respectively. Increasing the homology to these two consensus sequences might improve promoter activity, and vice versa. Changing “TATATA” to “TATAAT” in the −10 hexamer was predicted to strengthen the promoter of the hypothetical protein (A6M16_09815). RBS, ribosome-binding site.





Figure S2. Methyl methane sulfonate sensitivity assay shows no significant difference between DN13 and SS9-P10. Bacteria were treated with 0.1% methyl methane sulfonate for 20 min. After 10-fold serial dilution six times, 100 μl of dilutions were spread onto BHI agar for cell counting. Survival percentage was calculated as those in “Oxidative stress”. Non-parametric *t test* was used to analyze the data.

Figure S3. Phylogenetic analysis of SrtR in bacteria. Sequences were collected using BLASTp and only one sequence was selected for each species. MEGA6 was used to construct the phylogenetic tree. The LG+G model was selected for the construction of the Maximum Likelihood Tree as indicated by the software after calculation by “Find Best DNA/Protein Models”.

*Enterococcus ureilyticus* WP 069640634.1

*Enterococcus silesiacus* WP 071876746.1

*Enterococcus termitis* WP 086346106.1

*Enterococcus moraviensis* WP 010764260.1

*Enterococcus haemoperoxidus* WP 010763071.1

*Enterococcus quebecensis* WP 069634617.1

*Enterococcus plantarum* WP 111247736.1

*Enterococcus wangshanyuanii* WP 088271544.1

*Enterococcus phoeniculicola* WP 010767238.1

*Enterococcus rivorum* WP 069700024.1

*Lactobacillus fuchuensis* WP 106482876.1

*Enterococcus thailandicus* WP 071869213.1

*Enterococcus casseliflavus* WP 015510543.1

*Enterococcus gallinarum* WP 061053402.1

*Enterococcus faecium* WP 051154613.1

*Lactobacillus oligofermentans* WP 057889065.1

*Lactobacillus sakei* WP 035146844.1

*Streptococcus suis* CP015557

*Streptococcus plurextorum* WP 027972013.1

*Lactobacillus kalixensis* WP 057797064.1

*Lactobacillus amylovorus* WP 013437372.1

*Lactobacillus delbrueckii* WP 050952627.1

*Sharpea azabuensis* WP 033163429.1

*Eubacterium pyruvativorans* WP 090471585.1

*Clostridioides difficile* OJT77597.1

*Clostridium perfringens* WP 003482687.1

*Brevefilum fermentans* WP 087861420.1

*Eubacterium barkeri* WP 090244567.1

*Eubacterium aggregans* WP 090309887.1

*Lactobacillus pasteurii* WP 009559195.1

*Lactobacillus johnsonii* WP 069168771.1

*Carnobacterium pleistocenium* WP 035054246.1

*Carnobacterium divergens* WP 034568717.1

*Carnobacterium maltaromaticum* WP 056999977.1

*Shimazuella kribbensis* WP 028778633.1

*Lysinibacillus xylanilyticus* WP 100545602.1

*Bacillus wiedmannii* WP 097858240.1

*Lactobacillus paralimentarius* WP 056951262.1

*Lactobacillus mindensis* WP 057888120.1

*Lactobacillus kimchiensis* WP 057881171.1

0.2





Figure S4. SrtR is not involved in biofilm formation in *S. suis*. Values were expressed as mean percentages with standard deviation





Figure S5. Growth curve of DN13 and its passages. Overnight culture of bacteria were diluted with fresh BHI to an OD600 of 0.1 and cultured at 37 °C with shaking for 12 h. OD600 was monitored at 1-h intervals. Values were expressed as mean with range.





Figure S6. Bactericidal effect of mouse whole blood on DN13 and its passages. Mouse whole blood with 0.4% sodium citrate was used to kill *Streptococcus suis*. Bacteria were diluted to an OD600 of 0.1 and 100 μl of each dilution was added to 900 μl of sodium citrate-containing whole blood. The survival percentage at 120 and 0 min was calculated by cell counting after 10-fold serial dilutions and plating. Values were expressed as mean percentages with standard deviation
